# Assessment of prolonged proteasome inhibition through ixazomib‐based oral regimen on newly diagnosed and first‐relapsed multiple myeloma: A real‐world Chinese cohort study

**DOI:** 10.1002/cam4.7177

**Published:** 2024-04-30

**Authors:** Aijun Liu, Hong Yu, Rui Hou, Zunmin Zhu, Jun‐ling Zhuang, Li Bao, Zhenling Li, Lihong Liu, Luoming Hua, Yanping Ma, Da Gao, Arong Jin, Xiaohui Suo, Wei Yang, Yuansong Bai, Rong Fu, Deqiang Zheng, Wenming Chen

**Affiliations:** ^1^ Department of Hematology Beijing Chaoyang Hospital, Affiliated to Capital Medical University Beijing China; ^2^ Department of Hematology Tianjin Medical University General hospital Tianjin China; ^3^ School of Public Health Capital Medical University Beijing China; ^4^ Department of Hematology Henan Provincial people's hospital Zhengzhou China; ^5^ Department of Hematology Peking Union Medical Hospital Beijing China; ^6^ Department of Hematology Beijing Jishuitan Hospital Beijing China; ^7^ Department of Hematology China‐Japan Friendship Hospital Beijing China; ^8^ Department of Hematology The Fourth hospital, Affiliated to Hebei Medical University Shijiazhuang China; ^9^ Department of Hematology Affiliated Hospital of Hebei University Shijiazhuang China; ^10^ Department of Hematology Second hospital of Shanxi Medical University Taiyuan China; ^11^ Department of Hematology The Affiliated Hospital of Inner Mongolia Medical University Hohhot China; ^12^ Department of Hematology Inner Mongolia Autonomous Region People's Hospital Hohhot China; ^13^ Department of Hematology Han Dan Central Hospital Handan China; ^14^ Department of Hematology Sheng Jing Hospital of China Medical University Shenyang China; ^15^ Department of Hematology China‐Japan Union Hospital of Jilin University Changchun China

**Keywords:** duration of treatment, in‐class transition, proteasome inhibitor, real‐world community

## Abstract

**Objective:**

To evaluate the effectiveness, safety, and convenience of in‐class transition (iCT) from intravenous bortezomib‐based induction to ixazomib‐based oral regimens.

**Methods:**

This retrospective real‐world study was conducted in 16 Chinese hospitals between October 2017 and April 2023 and analyzed newly diagnosed (NDMM) and first‐line relapsed multiple myeloma (FRMM) patients who attained at least a partial response from bortezomib‐based induction therapy, followed by an ixazomib‐based oral regimen for 2 year or until disease progression or intolerable toxicity.

**Results:**

The study enrolled 199 patients, median age: 63 years old, male 55.4%, 53% as high risk (HR), and 47% as standard risk. Cytogenetic risk stratification by metaphase fluorescence in situ hybridization (M‐FISH), based on the Mayo Clinic risk stratification system. The median duration of total PI therapy was 11 months, with ixazomib‐based treatment spanning 6 months. At the 20‐month median follow‐up, 53% of patients remained on therapy. The 24‐month PFS rate was 84.3% from the initiation of bortezomib‐based induction and 83.4% from the start of ixazomib‐based treatment.

Overall response rate (ORR) was 100% post‐bortezomib induction and 90% following 6 cycles of the ixazomib‐based regimen. Based on the Sankey diagrams, 89.51% of patients maintained or improved their disease response after 2 cycles of iCT, 6 cycles (90.14%), and 12 cycles (80%). The HR level of Mayo was found to be a significant independent factor in a worse remission (hazard ratio (HR) 2.55; *p* = 0.033).

Ixazomib's safety profile aligned with previous clinical trial data, with 49% of patients experiencing at least one AE of any grade. The most common AEs included peripheral neuropathy, nausea and vomiting, diarrhea, thrombocytopenia, and granulocytopenia.

**Conclusion:**

In the real‐world Chinese MM population, NDMM and FRMM patients responded favorably to PI‐based continuous therapy, demonstrating substantial response rates. The ixazomib‐based iCT allows for sustained PI‐based treatment, offering promising efficacy and tolerable AEs.

## 1 BACKGROUND

Multiple myeloma (MM) is a hematologic malignancy marked by the clonal expansion of plasma cells. Proteasome inhibitor (PI)‐based therapies are currently recognized as the foundation for treating both transplant‐eligible and transplant‐ineligible newly diagnosed MM (NDMM), as well as relapsed MM. Substantial evidence indicates that persistent PI therapy confers survival benefits,^1^, ^2^, ^3^, ^4^ and a positive correlation exists between higher cumulative PI doses and enhanced overall survival (OS) in MM patients.^5^

However, implementing continuous PI treatment presents several obstacles in real‐world scenarios, often leading to a shorter duration of treatment (DOT) than what is typically seen in clinical trials. Several factors contribute to the less‐than‐ideal DOT in real‐world settings, including treatment‐related toxicities such as bortezomib‐induced peripheral neuropathy (PN), the burden associated with intravenous or subcutaneous administration that can adversely affect a patient's quality of life, advanced patient age accompanied by comorbidities, poor adherence to treatment protocols, and difficulties in accessing hospital care. Adverse events (AEs), such as PN, constitute one of the primary factors leading to the discontinuation of PI treatment. A comprehensive retrospective observational study from the United States revealed that the average DOT for patients with first relapsed MM (FRMM) receiving bortezomib and immunomodulatory imide drugs (IMiDs) was only 3.6 months, markedly shorter than the duration reported in randomized controlled trials (RCTs).^6^

A recent review reported that patients who received oral PI‐based regimens demonstrated comparable progression‐free survival (PFS) and time to next treatment (TTNT) in real‐world settings as compared to RCTs, suggesting that oral PIs will be the preferred long‐term PI choice in real‐world scenarios. Prompted by these findings, a study was initiated in the United States to evaluate the potential benefits of continuous PI treatment within a real‐world community context.^7^ Transplant‐ineligible newly diagnosed MM patients received a bortezomib‐based induction regimen spanning three treatment cycles. Subsequently, an ixazomib‐based regimen was administered once the patients achieved a minimum of stable disease. Updated evidence indicates that patients who were continuously administered with oral regimen attained deeper responses, accompanied by favorable safety, high adherence, and enhanced quality of life.^7^ The outcomes from other real‐world studies^8^, ^9^, ^10^, ^11^, ^12^, ^13^, ^14^, ^15^, ^16^ suggest that an ixazomib‐based oral regimen delivers similar efficacy to that reported in clinical trials,^17^ while also demonstrating robust tolerability.^9^, ^10^, ^17^, ^18^

In a study, the efficacy of iCT from induction therapy with intravenous bortezomib to all‐oral IRd regimen was compared to remaining on bortezomib‐based combination therapy. The findings suggest that the use of iCT can significantly improve the overall response rate (ORR) and prolong duration of treatment (DOT), indicating potential benefits for patients when compared to continuous bortezomib treatment.^19^ However, there is still a lack of evidence regarding the effectiveness, safety, and convenience of in‐class transition (iCT) from intravenous bortezomib‐based induction to ixazomib‐based oral regimens among the Chinese population, which we aimed to clarify in this multicenter real‐world study including a total of 199 patients with NDMM.

## 2 METHODS

### 2.1 Study design and participants

We conducted a multicenter, real‐world, retrospective study spanning from October 2017 to April 2023. A cohort of 199 MM patients was reviewed for the study, which included adult non‐transplant patients with newly diagnosed multiple myeloma MM and first‐line relapsed MM after lenalidomide maintenance, as characterized by the International Myeloma Working Group criteria. Patients who had received a minimum of 2 cycles of first‐line bortezomib‐based induction treatment and attained a partial response (PR) or better were deemed eligible. Furthermore, patients should have an Eastern Cooperative Oncology Group (or equivalent) performance status of 0–2, without grade ≥2 PN or grade 1 PN associated with pain.

Patient recruitment was executed across 16 hospitals in China, and the enrolled patients were treated with all‐oral ixazomib‐based regimens. These regimens encompassed ixazomib‐lenalidomide‐dexamethasone (IRD), ixazomib‐thalidomide‐dexamethasone (ITD), and ixazomib‐cyclophosphamide‐dexamethasone (ICD), which were administered until disease progression (PD) or onset of unacceptable toxicity. It was imperative for patients to continue ixazomib treatment to maintain their participation in the study. At the end of the treatment period or upon loss to follow‐up by patients' economic reason or COVID‐19 epidemic, patient assessments were performed by the clinicians.

The study protocol was conducted in accordance with the principles of the Declaration of Helsinki and approved by the Health Human Research Ethics Committee of Beijing Chaoyang Hospital, Affiliated to Capital Medical University (approval no. 2019‐327).

### 2.2 Outcomes and assessments

Data concerning demographics, blood chemistry, molecular cytogenetic subtype, treatment exposure, treatment duration, treatment response, adverse events, and survival outcome were systematically collected and analyzed. The primary endpoint was 2‐year progression‐free survival (PFS). PFS over a 2‐year period for each patient was computed, with PFS being defined as the duration from the initiation of bortezomib‐based induction therapy to the first documented instance of disease progression or death due to any cause. Key secondary end points were response rate, therapy duration, and adverse event (AE).

Additionally, we compiled and analyzed data related to response rates, duration of treatment, and safety profiles. Response and disease progression were assessed by clinicians following the response criteria established by the International Myeloma Working Group. Safety profiles of ixazomib‐based oral regimens were evaluated through ongoing monitoring of AEs during the course of the study. The severity of toxicity was categorized in accordance with the National Cancer Institute's Common Terminology Criteria for Adverse Events (version 4.03).

### 2.3 Statistical analysis

Continuous variables were represented using means and standard deviations, or medians and quartiles, while categorical variables were described using frequencies and percentages. The missforest approach was applied to impute missing records, accommodating both categorical and continuous variables. We utilized Sankey diagrams to visualize the evolution or transition from one state to another or from one time point to another. In our study, these diagrams were particularly employed to depict disease response trajectories across different treatment cycles. The objectives of the Sankey diagrams were to ascertain the proportion of patients exhibiting varying degrees of efficacy during the second, fourth, sixth, and eighth treatment cycles. The Cox proportional hazard model was used to analyze the survival data. To identify factors correlated with disease progression or death, the stepwise variable selection procedure with Cox regression was applied. The estimates and 95% confidence intervals (CIs) of the hazard ratio (HR) were calculated by applying Cox regression. A two‐tailed *p* value < 0.05 was considered statistically significant. Survival probabilities were estimated using Kaplan–Meier curves, and differences in PFS between groups were compared using the Log‐Rank test. The R package networkD3 was utilized to generate the Sankey diagrams, while the missforest approach was facilitated by the R package missForest. All remaining statistical analyses were conducted using SAS (version 9.4, SAS Institute, Cary, NC).

## 3 RESULTS

### 3.1 Patients and treatment

As of April 2023, a total of 199 patients (82% NDMM; 18% FRMM) had been enrolled across 16 study sites and had received at least one dose of the ixazomib‐based study drug regimen. Baseline demographic information is provided in Table 1. The median patient age was 63 years, with 43% of patients aged ≥65 years and 7% aged ≥75 years. The cohort comprised 55% men. Thirty‐nine percent were categorized as International Staging System (ISS) stage III, and 21% were Revised International Staging System (R‐ISS) stage III. Based on the Mayo Clinic risk stratification, 53% (69/129) of patients were at high risk, and 47% (60/129) were at standard risk.

**TABLE 1:** Baseline characteristics of patients.*

Characteristic	Ixazomib‐based regimens (*n* = 199)	
Median age, years (range)	63	(39 ~ 87)
Age ≥ 65 years, *n* (%)	85	(43)
Age ≥ 75 years, *n* (%)	14	(7)
Male, *n* (%)	109	(55)
NDMM, *n* (%)	164	(82)
FRMM, *n* (%)	35	(18)
DS stage at initial diagnosis, *n* (%)		
I	10	(5)
II	24	(12)
III	146	(73)
Unknown	19	(10)
ISS stage at initial diagnosis, *n* (%)		
I	27	(14)
II	83	(41)
III	77	(39)
Unknown	12	(6)
R‐ISS stage at initial diagnosis, *n* (%)		
I	9	(5)
II	67	(34)
III	42	(21)
Unknown	81	(40)
Mayo clinic risk stratification, *n* (%)		
Standard risk	60	(30)
High risk	69	(35)
Unknown	70	(35)
Type of myeloma at initial diagnosis, *n* (%)		
IgG	87	(44)
IgA	50	(25)
IgD	5	(3)
Light chain	46	(23)
Non‐secretory	2	(1)
Unknown	9	(4)
Serum creatinine, *n* (%)		
<177 μmol/L	127	(64)
≥177 μmol/L	47	(24)
Unknown	25	(12)
Extramedullary disease *n* (%)		
Yes	16	(8)
No	126	(64)
Unknown	57	(28)
Induction regimen at the time of iCT to ixazomib, *n* (%)		
VRD	79	(40)
VTD	13	(7)
VD	19	(10)
VCD	50	(25)
VAD	21	(10)
Others	17	(8)

* At enrollment or initial diagnosis conditions are listed by preferred term.

Abbreviations: DS, Durie‐Salmon; iCT, in‐class transition; ISS, International Staging System; R‐ISS, Revised International Staging System; VAD, bortezomib‐doxorubicin‐dexamethasone.; VCD, bortezomib‐cyclophosphamide‐dexamethasone; VD, bortezomib‐dexamethasone; VRD, bortezomib‐lenalidomide‐dexamethasone; VTD, bortezomib‐thalidomide‐dexamethasone.

All patients underwent a median of 4 cycles (range 1–10 cycles) of bortezomib‐based therapy. The most prevalent induction regimen at the time of transition to ixazomib‐based therapy was a combination of bortezomib and IMiDs‐based regimens (40% bortezomib‐lenalidomide‐dexamethasone [VRD], 7% bortezomib‐thalidomide‐dexamethasone [VTD]). (Table 1). The administration of bortezomib, given at a most common dose of 1.3 mg/m^2^, was once or twice a week, depending on patient performance status. Subsequently, all patients transitioned to a median of 4 cycles (range 1–20 cycles) of ixazomib‐based therapy. The transitional regimens included IRD (145/199), ITD (18/199), and ICD (36/199). The majority of patients received ixazomib and IMiD‐based regimens (163/199). At the data cut‐off point, 106 patients (53%) remained on therapy while 93 (47%) had discontinued treatment, with the reasons being disease progression (15%, 30/199) and toxicity (9%, 18/199), notably PN (9%, 18/199). Of those patients with significant PN, 89% (8/9) had exhibited prominent PN during bortezomib‐based treatment.

### 3.2 Efficacy profiles

During a median follow‐up period of 20 months, 20 patients (10.05%) died due to any cause, and 30 patients (15.08%) exhibited disease progression during ixazomib treatment (median, 4 cycles; range, 2–8). The 2‐year PFS rate was 84.3% starting from the initiation of bortezomib‐based induction and 83.4% from the commencement of ixazomib‐based treatment. The median duration of total PI therapy spanned 11 months (range, 1–43 months), whereas that of ixazomib‐based regimen was 6 months (range, 1–20 months).

#### 3.2.1 The results of Sankey diagrams showed that iCT deepens the patients' remission

**FIGURE 1:**
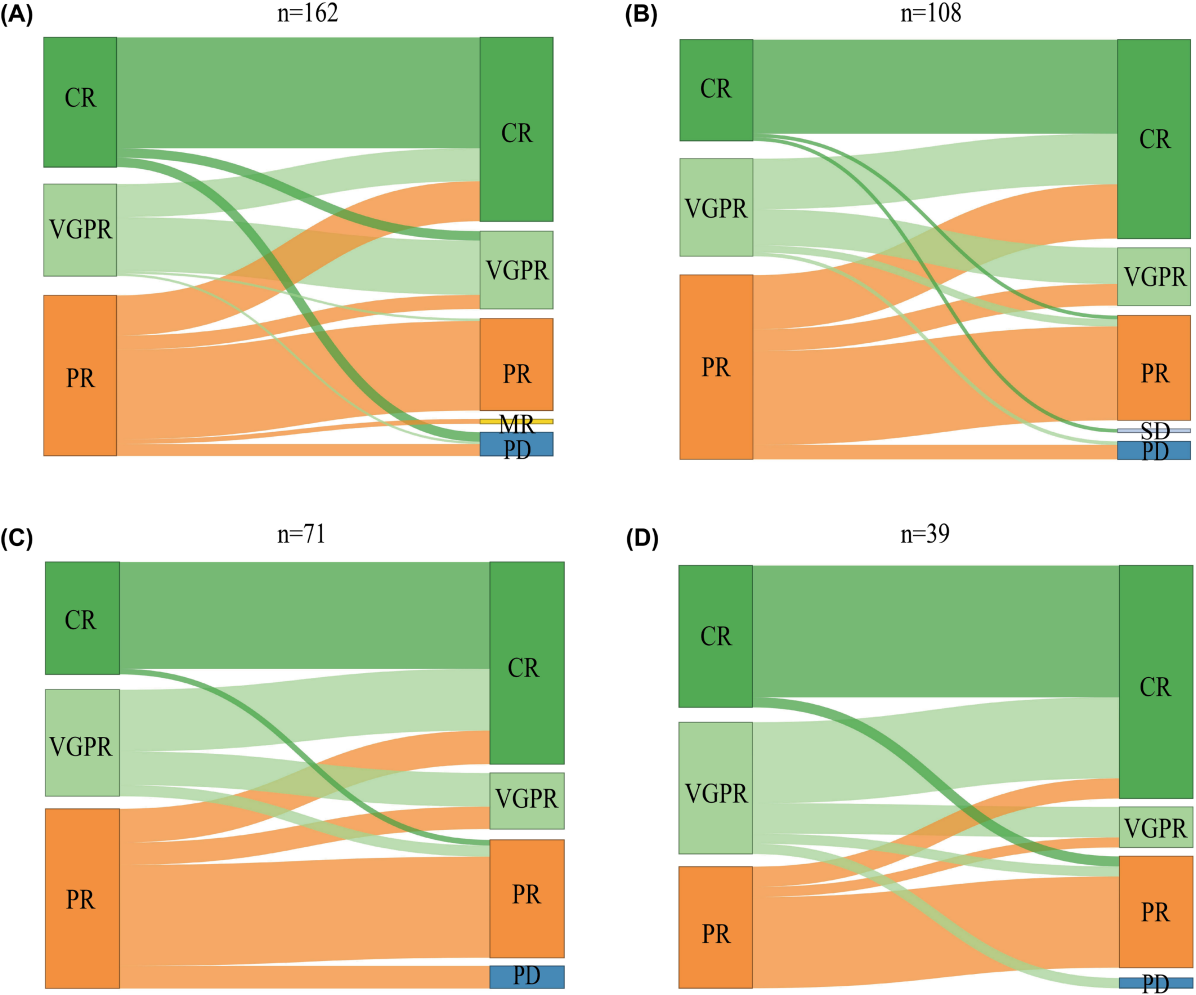
Sankey diagram was presented to illustrate the disease response from pre‐medication to the second (A), fourth (B), sixth (C), and eighth (D) medication cycles following iCT to ixazomib‐based therapy. CR, complete response; MR, Minimal response; PD, progressed disease; PR, partial response; VGPR, very good partial response.

**FIGURE 2:**
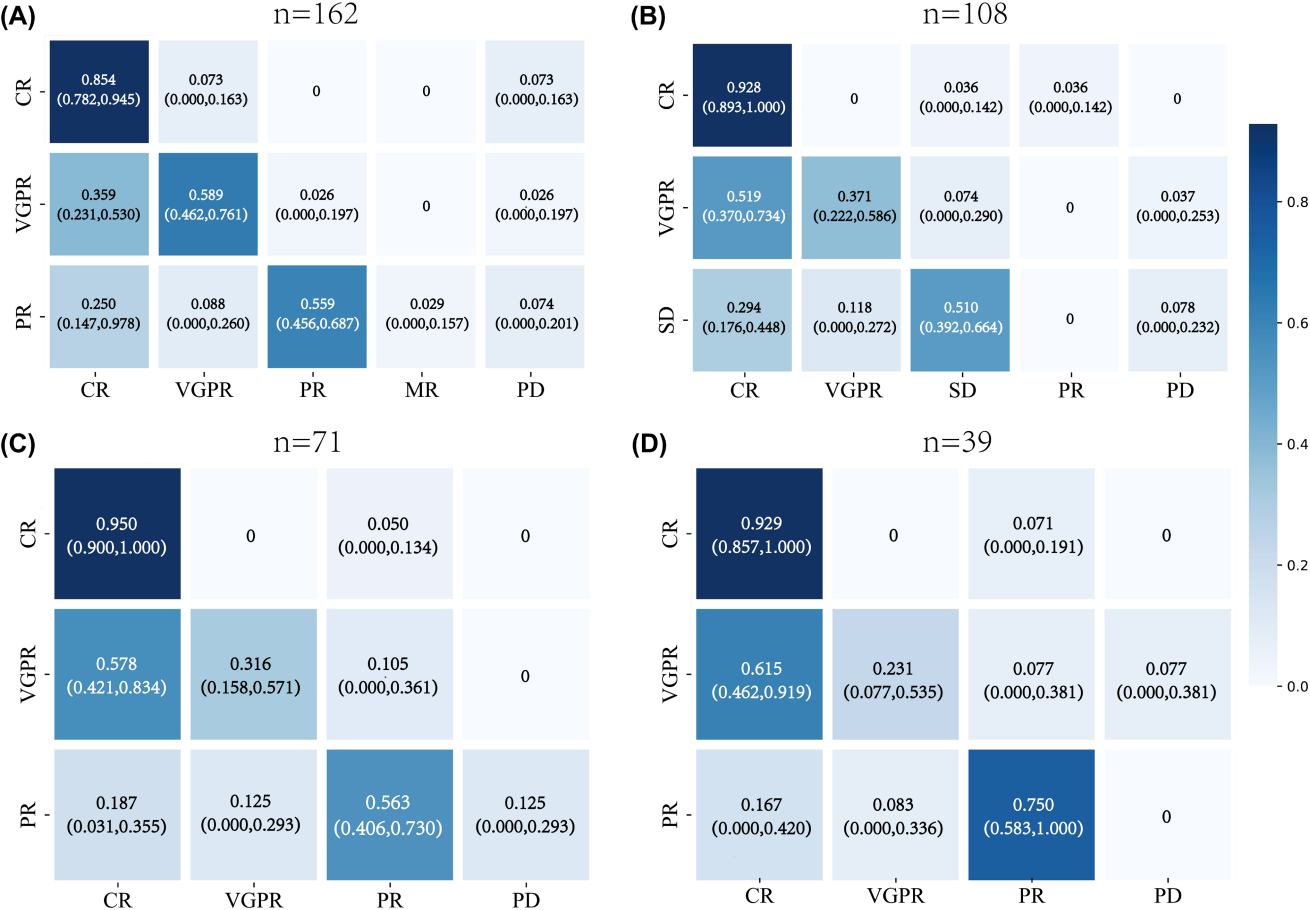
Confusion matrix was presented to illustrate the disease response from pre‐medication to the second (A), fourth (B), sixth (C), and eighth (D) medication cycles following iCT to ixazomib‐based therapy. CR, complete response; MR, Minimal response, PD, progressed disease; PR, partial response; VGPR, very good partial response.

During bortezomib‐based induction, the best responses were as follows: stringent complete response (sCR) in 9% (18/199), CR in 24% (47/199), very good partial response (VGPR) in 27% (54/199), and PR in 40% (80/199) of patients. Following the iCT to ixazomib‐based therapy, the efficacy was evaluated in 162 patients. A Sankey diagram demonstrating the disease response from pre‐medication to various medication cycles is depicted in Figure 1. For example, after two treatment cycles, 85.4% (95% CI: 78.2%–94.5%) of patients sustained their original status, while 7.3% (95% CI: 0.0%–16.3%) transitioned to VGPR and PD, respectively. In the VGPR group, 58.9% (95% CI: 78.2%–94.5%) maintained VGPR, 35.9% (95% CI: 23.1%–53.0%) shifted to CR, and 2.6% (95% CI: 0.0%–19.7%) reverted to PR and PD. In the PR group, 89.7% improved to PR or better, while 10.3% exhibited disease progression to minimal response (MR) (2.9%, 95% CI: 0.0%–15.7%) and PD (7.4%, 95% CI: 0.0%–20.1%). Overall, 89.51% (145/162) of patients retained the same disease response or transitioned to a superior disease response after 2 cycles (Figure 1). Notably, over 80% of patients maintained or improved their disease responses after the 4th (91.51%, 97/106), 6th (90.14%, 64/71), and 8th (90.14%, 36/39) (Figure 2).

#### 3.2.2 Both standard‐risk MM and high‐risk MM patients can benefit from iCT, either with IRD/ITD or ICD regimens

**FIGURE 3:**
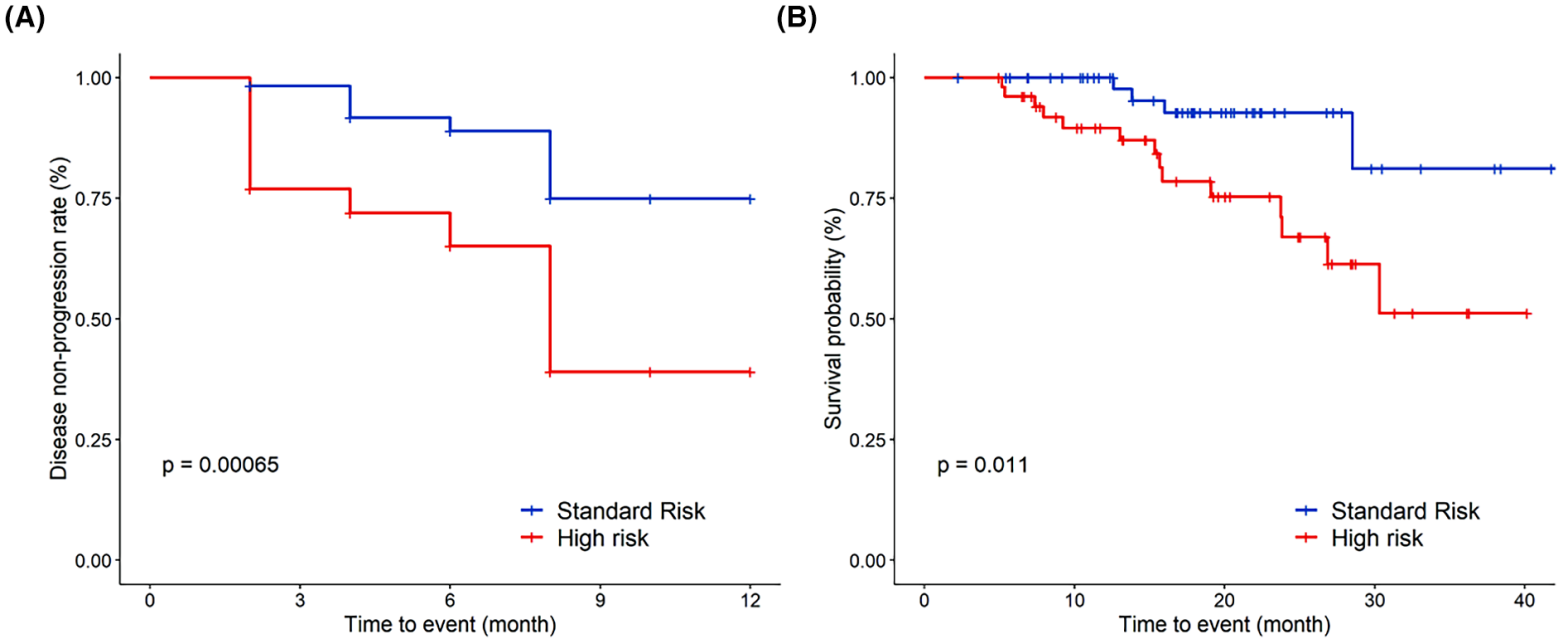
Investigator‐assessed best overall responses after bortezomib‐based induction and after in‐class transition (iCT) to ixazomib‐based induction by Mayo risk stratification. Kaplan–Meier curves were used to depict the disease non‐progression rate (A) and survival probability (B) of the high‐risk group and standard‐risk group.

Additionally, the depth of remission showed enhancement for both standard‐risk MM and high‐risk HR MM patients. The overall response rate (ORR) following bortezomib‐based induction was 100%. In the standard‐risk MM cohort, 7%, 34%, 25%, and 34% achieved sCR, CR, VGPR, and PR as their best response, respectively (Figure S1). For the high‐risk MM cohort, these responses were achieved by 11%, 19%, 31%, and 39%, respectively. Upon iCT to ixazomib‐based therapy, the ORR for the standard‐risk group was 96%. In this group, the rates of sCR and CR increased to 18% and 43%, respectively, while the rates of VGPR and PR decreased to 14% and 21%, respectively. For the high‐risk group, ORR was 82%, with sCR and CR rates increasing to 17% and 20%, respectively, and VGPR and PR rates decreasing to 26% and 19%, respectively. For the group with unknown risk, the ORR remained at 100% before and after the iCT to ixazomib‐based therapy, with sCR rate increasing from 12% to 23%, CR rate increasing from 21% to 39%, and PR rate decreasing from 50% to 22% post‐iCT (Figure S1). During follow‐up, disease progression occurred in 8 of 57 in the Mayo standard‐risk group and in 22 of 52 in the Mayo high‐risk group. The incidence of disease progression was significantly different between the two groups (Figure 3A). Similarly, there was a significant difference in survival between the two groups (Figure 3B).

For the 162 patients iCT to the IRD/ITD regimen, the median duration of total PI therapy was 12 months, whereas the median duration of the ixazomib‐based regimen was 6 months. The best response rates during bortezomib‐based induction were 10%, 25%, 25%, and 40% for sCR, CR, VGPR, and PR, respectively. After initiation of ixazomib‐based regimens, the best responses transitioned to 20%, 35%, 18%, and 21% for sCR, CR, VGPR, and PR, respectively, leading to an ORR of 94% (Figure S2).

Among the 30 patients who exhibited PD during ixazomib treatment, FISH results were available for 19 patients (high‐risk *n* = 5; standard‐risk *n* = 14). Throughout the course of ixazomib‐based treatment, eight patients achieved sCR/CR, of which two were classified as high‐risk and five lacked FISH results. Five patients attained VGPR as their best response, with two of them being high‐risk. A further nine patients achieved PR; among these, four were high‐risk, and five lacked FISH results. The remaining six high‐risk patients all demonstrated disease progression.

### 3.3 Cox regression analysis results

After excluding participants who completed less than 2 cycles of treatment and those who did not undergo FISH testing, we analyzed data from 109 patients. Baseline demographic information is provided in Table S1. The mean age at diagnosis was 62.40 years, and 47.71% of the cohort were women. Initial disease responses were as follows: 47 participants (43.12%) demonstrated a PR, 29 (26.60%) achieved a VGPR, and 33 (30.28%) recorded a CR. According to the Mayo Clinic risk stratification, 53 subjects (48.62%) were categorized as high‐risk, while 85 subjects (77.98%) were classified as stage III under the Durie‐Salmon (DS) system. During the follow‐up period (median, 540 days; range, 352–720 days), 18 patients (16.51%) died due to any cause, and 30 patients (27.52%) exhibited disease progression during ixazomib treatment (median, 4 cycles; range, 2–8).

Stepwise Cox regression identified four variables related to disease progression: Mayo risk stratification, the presence of plasmacytoma, deletion of 17p (del (17p)), and disease response at the second cycle. The high‐risk status according to the Mayo Clinic risk stratification emerged as a significant independent predictor of poorer disease response (HR, 2.55; 95% CI, 1.08 to 6.04, *p* = 0.033) in the adjusted Cox regression model (Table 2). Additionally, plasmacytoma and del (17p) were risk factors, but they did not reach statistical significance, with HRs of 2.07 (95% CI, 0.74–5.79; *p* = 0.164) and 1.92 (95% CI, 0.75–4.87, *p* = 0.172), respectively. Cox analysis also revealed that a better disease response at the second cycle was associated with a reduced risk of disease progression (HR, 0.489; 95% CI, 0.358–0.668; *p* < 0.0001). When evaluating predictors of mortality, stepwise Cox regression identified seven variables. Notably, a high‐risk status according to the Mayo Clinic risk stratification (HR, 4.97; 95% CI, 1.27–19.46, *p* = 0.021) and the presence of plasmacytoma (HR, 7.18; 95% CI, 1.43–36.15; *p* = 0.017) were associated with an increased risk of death. Moreover, disease response at the second cycle (HR, 0.59; 95% CI, 0.42–0.82; *p* = 0.002) and serum albumin level (HR, 0.90; 95% CI, 0.82–0.99; *p* = 0.032) were also significantly associated with the risk of death (Table 2).

**TABLE 2:** Cox proportional hazards models of disease progression and death.

Variable		All	Disease progression			Death		
			HR	95%CI	*p*	HR	95%CI	*p*
Mayo	0	57 (52)						
	1	52 (48)	2.55	1.08–6.04	0.034	4.97	1.27–19.46	0.021
Plasmacytoma	0	100 (92)						
	1	9 (8)	2.07	0.74–5.79	0.164	7.178	1.43–36.15	0.017
Del 17	0	96 (88)						
	1	13 (12)	1.92	0.75–4.87	0.172	2.77	0.63–12.10	0.176
Disease response of the second cycle	Median (range)	4 (3–5)	0.49	0.36–0.67	<0.0001	0.59	0.42–0.82	0.002
t (11:14)	0	95 (87)						
	1	14 (13)				0.19	0.03–1.14	0.069
Albumin	Median (range)	34 (31–39)				0.90	0.82–0.99	0.032
Hemoglobin	Median (range)	101 (82–117)				1.03	0.99–1.06	0.058

Abbreviations: CI, confidence interval; HR, hazard ratio.

### 3.4 Safety profiles

The safety profile of the ixazomib‐based regimens is presented in Table 3. AEs of any grade were reported in 49% of patients. The most frequently encountered AEs of grade ≤2 included PN (28%), nausea and vomiting (28%), and diarrhea (8%). AEs of grade 3/4 comprised agranulocytosis, diarrhea, and rash, each reported in 2% of patients. Of all the patients enrolled in the study, 9% discontinued the ixazomib‐based regimen due to AEs, with PN (*n* = 9), incomplete intestinal obstruction (*n* = 3), diarrhea (*n* = 2), nausea and vomiting (*n* = 1), agranulocytosis (*n* = 1), and rash (*n* = 2) being the primary reasons for discontinuation.

**TABLE 3:** Treatment‐emergent adverse events (TEAEs) during ixazomib‐based treatment.

TEAE, *n* (%)	All (*N* = 199)			
	Any Grade		Grade 3/4	
Peripheral neuropathy	56	(28)	1	(1)
Nausea and vomiting	55	(28)	1	(1)
Diarrhea	16	(8)	3	(2)
Incomplete intestinal obstruction	3	(2)	0	(0)
Constipation	8	(4)	0	(0)
Agranulocytosis	8	(4)	4	(2)
Rash	6	(3)	3	(2)
Thrombocytopenia	6	(3)	2	(1)
Fatigue	4	(2)	0	(0)
Weakness	2	(1)	0	(0)
Anorexia	2	(1)	0	(0)
Abdominal distension	2	(1)	0	(0)
Edema	2	(1)	0	(0)

## 4 DISCUSSION

In our study, Sankey diagrams were conducted to characterize the trajectories of disease response from an initial treatment to different cycles. Based on the current results, 89.51% (145/162) of patients maintained or improved their disease response after 2 cycles of iCT. After 4 cycles (91.51%, 97/106), 6 cycles (90.14%, 64/71), and 8 cycles (90.14%, 3/39), more than 90% of patients continued to maintain their disease response or showed improvement. These findings indicate that iCT deepens the patients' remission, which is consistent with the US‐MM6 study, where iCT to all‐oral IRd regimen led to a continuous increase in overall response rate (ORR) and complete response (CR) rate, with the ORR improving from 62% to 80%.^20^ Furthermore, our study showed the 2‐year PFS rate of 84.3% starting from the initiation of bortezomib‐based induction and 83.4% from the commencement of ixazomib‐based treatment, which is superior to the data of US‐MM6 study (71% from the commencement of ixazomib‐based treatment).^20^

The high‐risk level of Mayo was found to be a significant independent factor of a worse disease response. Multiple studies have shown that the high‐risk level of Mayo is a prognostic factor for disease progression.^21^, ^22^, ^23^ Our study found that, after iCT, both standard‐risk and high‐risk patients showed an improvement in the ≥VGPR rate, similar to the results of the TOURMALINE‐MM1 study. The TOURMALINE‐MM1 study showed that IRd can significantly prolong PFS in patients with high‐risk cytogenetics and provide clinical benefits for those with increased non‐canonical NF‐κB pathway activity.^24^, ^25^

Furthermore, our analysis suggested that an early treatment response is a prognostic determinant for disease progression. MM is characteristically marked by pronounced biological and clinical heterogeneity. Presently, several studies have delved into the correlation between drug response dynamics and the prognosis of MM, revealing that the depth of remission and the time to achieving optimal remission are intimately linked with clinical outcome.^26^, ^27^, ^28^ The deeper the remission, the more favorable the patient prognosis, aligning with our results. Our data indicate that an improved disease response after 2 cycles of treatment acts as an independent prognostic factor for PFS. This suggests that both the depth of remission and the timeframe to achieve the optimal depth of remission collectively influence the survival outcome in MM.

In contrast to the US‐MM6 study, which solely recruited transplant‐ineligible NDMM patients, our study broadened the cohort to include patients with FRMM. Our study found that the median PFS of patients who iCT to ixazomib‐based regimens as initial treatment and those in the FRMM group were not reach, suggesting that iCT may allow these patients to achieve continuous PI treatment in clinical practice. Additionally, we conducted an analysis of the ixazomib‐based regimens, there was no statistically significant difference in PFS between the IRd/ITd and ICd (ixazomib, cyclophosphamide, dexamethasone) groups (*p* = 0.442). Our research findings suggest that continuous treatment with an ixa‐based regimen can provide benefits for patients with multiple myeloma, which is consistent with real‐world research results from other countries such as Turkey, Japan, South Korea, the Czech Republic, and several global multicenter studies.^18^, ^29^, ^30^, ^31^, ^32^

The safety profile of ixazomib‐based regimens in our study, evaluated at the data cutoff, aligns with the US‐MM6 study and preceding clinical trials. Gastrointestinal events and PN emerged as the most common treatment‐emergent adverse events (TEAEs) post‐iCT. Among the 59 patients who presented with PN prior to iCT, 54% were of grade 2–3. Subsequent to iCT, only a single case (1%) developed grade 3/4 PN, and nine patients (5%) discontinued therapy due to PN, eight of whom experienced PN prior to iCT. These findings resonate with past research, such as the TOURMALINE‐MM1 study, wherein the incidence of grade 3/4 PN during continuous IRD treatment was 2%, paralleling the RD group.^17^

## 5 CONCLUSION

The iCT offers a potential avenue for sustained PI treatment in real‐world scenarios. Ixazomib, an oral PI, enhances efficacy and safety through early iCT following bortezomib usage, fostering continuous PI therapy in transplant‐ineligible NDMM and FRMM patients. However, it is noteworthy that our study's median patient age was lower than that of the US‐MM6 study, and we did not gather quality‐of‐life data from patients. Future investigations are warranted to address these limitations.

## AUTHOR CONTRIBUTIONS

**Aijun Liu:** Writing – original draft (equal). **Hong Yu:** Data curation (equal); writing – review and editing (equal). **Rui Hou:** Formal analysis (equal); investigation (equal); writing – review and editing (equal). **Zunmin Zhu:** Resources (equal); writing – review and editing (equal). **Jun‐ling Zhuang:** Resources (equal); writing – review and editing (equal). **Li Bao:** Resources (equal); writing – review and editing (equal). **Zhenling Li:** Resources (equal); writing – review and editing (equal). **Lihong Liu:** Resources (equal); writing – review and editing (equal). **Luoming Hua:** Resources (equal); writing – review and editing (equal). **Yanping Ma:** Resources (equal); writing – review and editing (equal). **Da Gao:** Data curation (equal); validation (equal). **Arong Jin:** Investigation (equal); supervision (equal). **Xiaohui Suo:** Formal analysis (equal); validation (equal). **Wei Yang:** Investigation (equal); validation (equal). **Yuansong Bai:** Formal analysis (equal); validation (equal). **Rong Fu:** Resources (equal); writing – review and editing (equal). **Deqiang Zheng:** Data curation (equal); formal analysis (equal); writing – review and editing (equal). **Wenming Chen:** Conceptualization (equal); resources (equal); writing – review and editing (equal).

## CONFLICT OF INTEREST STATEMENT

All authors declare no competing interests.

## ETHICAL APPROVAL

The study protocol was conducted in accordance with the principles of the Declaration of Helsinki and approved by the Health Human Research Ethics Committee of Beijing Chaoyang Hospital, Affiliated to Capital Medical University (approval no.2019‐327). Written informed consent was obtained from the participates.

## Supporting information


Appendix S1.

## Data Availability

All data generated or analyzed during this study are included in this published article and its supplementary information files.
