# Supplementary material for: Assessment of prolonged proteasome inhibition through ixazomib‐based oral regimen on newly diagnosed and first‐relapsed multiple myeloma: A real‐world Chinese cohort study
Source: Cancer Med. 2024 Apr 30;13(9):e7177. doi: 10.1002/cam4.7177 (PMC11058688; doi:10.1002/cam4.7177)
Supplement: Supplementary file 1 — Appendix S1. [file CAM4-13-e7177-s001.docx]

***Supplementary Information***


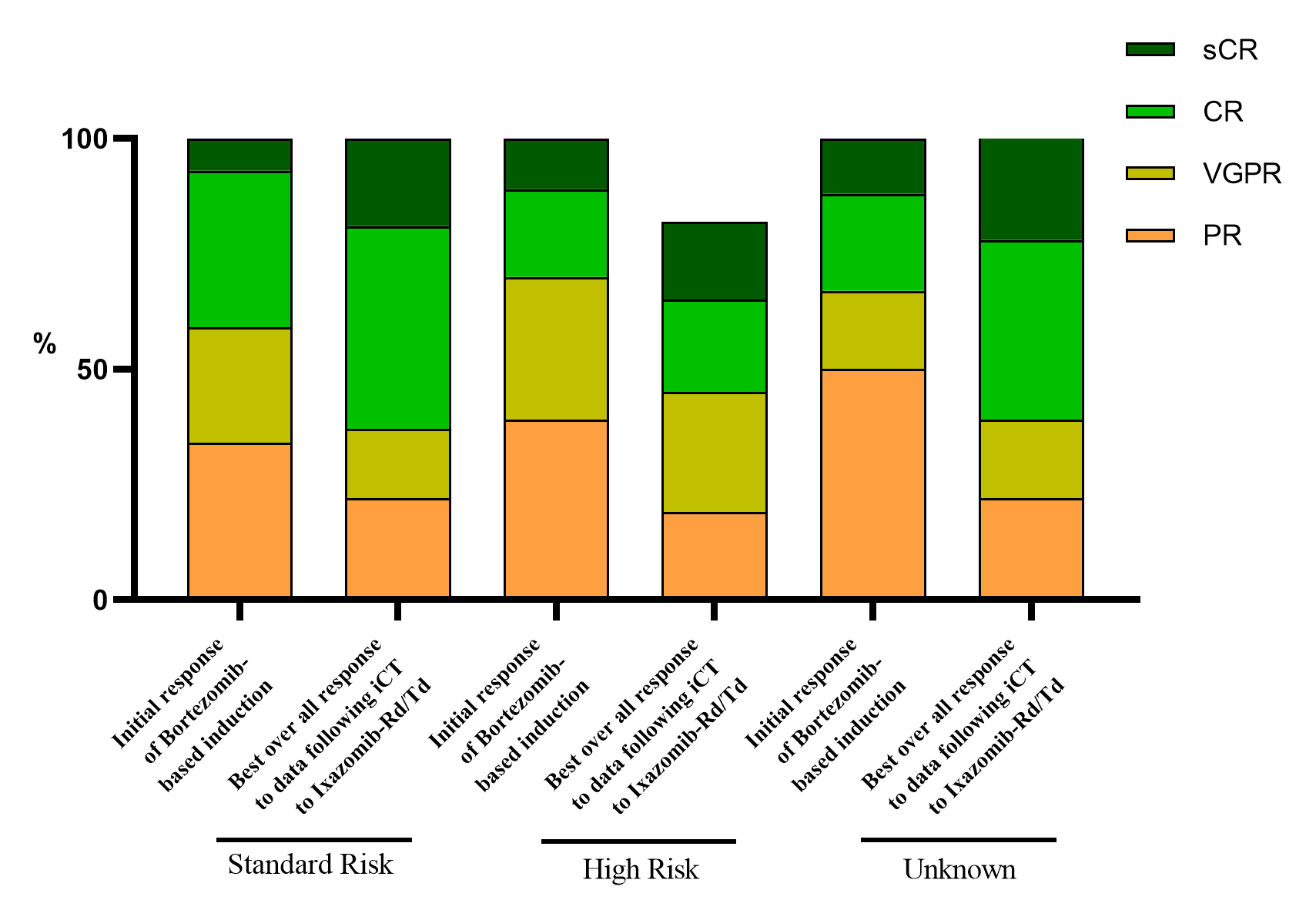


Supplementary Figure 1. The final responses were assessed after bortezomib-based induction and subsequent transition to ixazomib-based regimens for the standard-risk group, high-risk group, and unknown group, respectively.


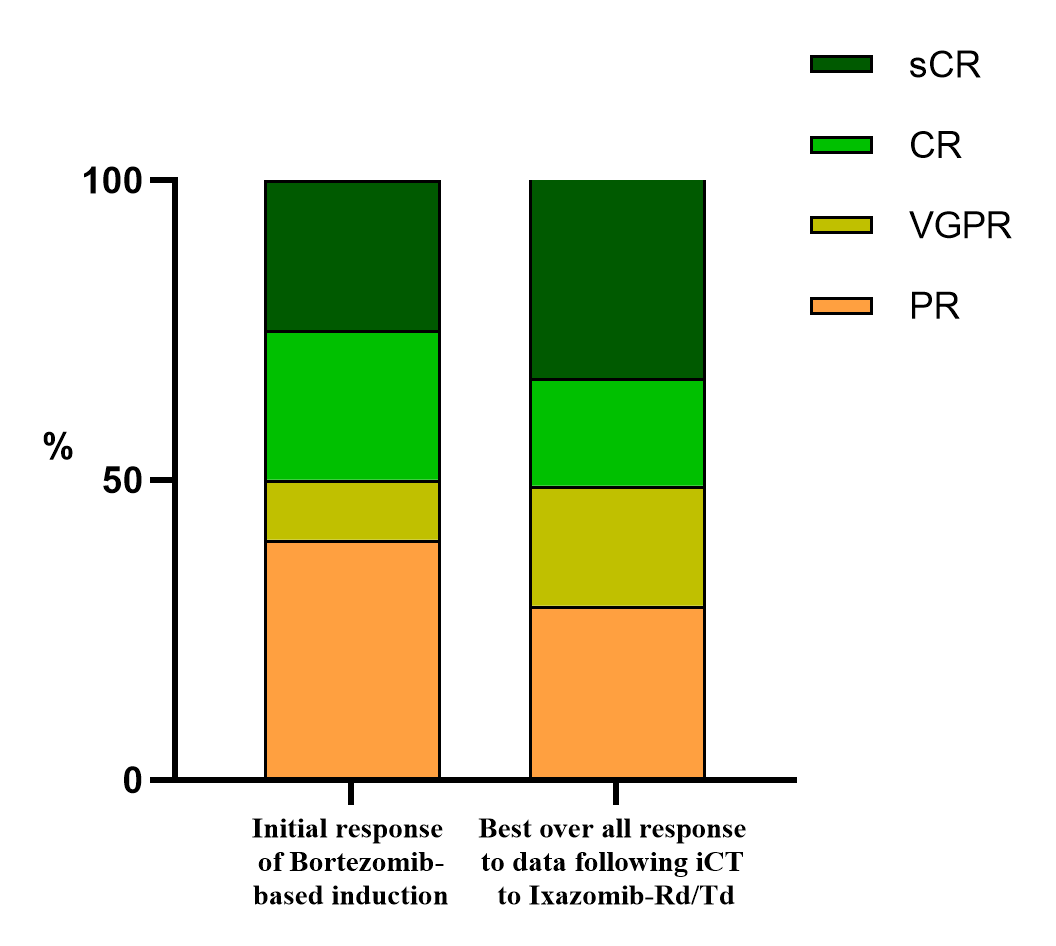


Supplementary Figure 2. The final responses were evaluated after bortezomib-based induction and subsequent transition to ixazomib-based regimens for patients in the IRD/ITD group.

Supplementary Table 1. Baseline characteristics of the patients included in the analysis (n= 109)

| **Characteristic** | **Female (N = 52)** | **male (N = 57)** | ***P* value** |
| --- | --- | --- | --- |
| Age at diagnosis, (year) | 62.90 (7.36) | 61.95 (7.65) | 0.508 |
| DS stage at diagnosis, n (%) |  |  | 0.875 |
| I | 3 (5.77) | 4 (7.02) |  |
| II | 8 (15.38) | 7 (12.28) |  |
| III | 41 (78.85) | 46 (80.70) |  |
| MAYO, n (%) |  |  |  |
| Low risk group | 27 (51.92) | 30 (52.63) | 0.999 |
| High risk group | 25 (48.08) | 27 (47.37) |  |
| Hemoglobin, (mean (SD)) | 100.65 (21.52) | 95.01 (23.66) | 0.197 |
| Platelets, (mean (SD)) | 171.54 (56.05) | 186.52 (86.63) | 0.291 |
| Albumin, (mean (SD)) | 35.96 (6.43) | 33.82 (12.27) | 0.262 |
| Lactate dehydrogenase, (mean (SD)) | 165.33 (45.59) | 189.96 (69.89) | 0.033 |
| A Lkaline Phosphatase, (mean (SD)) | 76.42 (35.85) | 77.42 (33.36) | 0.881 |
| Serum calcium, (mean (SD)) | 2.41 (0.38) | 2.39 (0.45) | 0.759 |
| Serum creatinine, (median [IQR]) | 71.50 [57.00, 95.40] | 75.50 [64.00, 125.00] | 0.122 |
| B2MG, (median [IQR]) | 4.97 [3.17, 10.62] | 5.48 [3.92, 9.89] | 0.404 |
| circle_before ixazomib, (mean (SD)) | 4.79 (2.52) | 4.43 (2.02) | 0.413 |
| circle_ixazomib, (mean (SD)) | 5.78 (3.96) | 6.17 (4.37) | 0.626 |
| Relapse, n (%) | 9 (17.31) | 11 (19.30) | 0.984 |
| Renal function, n (%) | 41 (78.85) | 39 (68.42) | 0.311 |
| Plasmacytoma, n (%) | 3 (5.77) | 6 (10.53) | 0.580 |
| 1q21^a^, n (%) | 14 (26.92) | 19 (33.33) | 0.604 |
| del(17p)^a^, n (%) | 6 (11.54) | 7 (12.28) | 0.999 |
| t (11;14) ^a^, n (%) | 4 (7.69) | 4 (7.69) | 0.212 |
| t (4;14) ^a^, n (%) | 10 (17.54) | 10 (17.54) | 0.715 |
